# Initial Experience with Sildenafil, Bosentan, and Nitric Oxide for Pediatric Cardiomyopathy Patients with Elevated Pulmonary Vascular Resistance before and after Orthotopic Heart Transplantation 

**DOI:** 10.1155/2010/656984

**Published:** 2010-03-10

**Authors:** Babak Daftari, Juan Carlos Alejos, Gregory Perens

**Affiliations:** UCLA Medical Center, Mattel Children's Hospital, Los Angeles, CA 90025, USA

## Abstract

*Background*. Although pulmonary hypertension complicating dilated cardiomyopathy has been shown to be a significant risk factor for graft failure after heart transplantation, the upper limits of pulmonary vascular resistance (PVR) that would contraindicate pediatric heart transplantation are not known. *Methods*. A retrospective review of all pediatric orthotopic heart transplant (OHT) performed at our institution from 2002 to 2007 was performed. Seven patients with PVR > 6 Wood's units (WU) prior to transplant were compared pre- and postoperatively with 20 matched controls with PVR < 6 WU. All pulmonary vasodilator therapies used are described as well as outcomes during the first year posttransplant. *Results*. The mean PVR prior to transplantation in the 7 study cases was 11.0 ± 4.6 (range 6–22) WU, compared to mean PVR of 3.07 ± 0.9 WU (0.56–4.5) in the controls (*P* = .27 × 10^−6^). All patients with elevated PVR were treated pre-OHT with either Sildenafil or Bosentan. Post-OHT, case patients received a combination of sildenafil, iloprost, and inhaled nitric oxide. All 7 case patients survived one year post-OHT, and there was no statistical difference between cases and controls for hospital stay, rejection/readmissions, or graft right ventricular failure. Mean PVR in the cases at one and three months post-OHT was not significantly different between the two groups. Only one of the cases required prolonged treatment with iloprost after OHT. *Conclusions*. A PVR above 6 WU should not be an absolute contraindication to heart transplantation in children.

## 1. Introduction

Children with cardiomyopathies often develop elevated pulmonary vascular resistance due to chronic left ventricular dysfunction and elevated left atrial pressures. Elevated pulmonary pressures add a significant risk factor to heart grafts exposed to donor brain death and ischemia during transport to the recipient. Preexisting pulmonary hypertension has been shown in numerous studies to be a risk factor for increased mortality after (OHT) [1–7]. Previous retrospective studies on larger cohorts of pediatric patients have shown up to 75% incidence of right ventricular failure with 15% mortality after transplant in patients with pre-OHT PVR > 6 Wood's Units (WU), compared to a 20% incidence of right heart failure in those without preoperative elevated PVR [8, 9]. Case reports in both the pediatric and adult populations have shown that treatment pre-OHT with therapies for pulmonary hypertension can lower PVR and allow for safe transplantation [10–12]. 

A number of treatment modalities are now available to treat pulmonary arterial hypertension. Intravenous nitroprusside and prostacyclin, and inhaled nitric oxide have proven valuable for both assessment and perioperative treatment in heart transplant candidates [13–15]. Unfortunately, many cardiomyopathy patients may need treatment for pulmonary hypertension for extended periods both before and after heart transplantation. The newer oral treatments for pulmonary hypertension, bosentan and sildenafil, have not been well evaluated in pediatric patients. In the adult population, bosentan has been shown to lower PVR to levels acceptable for heart transplantation, and one case report has shown that these patients can go on to be successfully transplanted [12]. Additionally, bosentan has been shown to significantly improve six-minute walk distances in adults with pulmonary hypertension [16]. Clearly, there is a significant advantage of orally administered medications for the chronic care of transplant patients. Many are on the waiting list of pre-OHT as outpatients and require home medication at hospital discharge post-OHT. Bosentan is an oral nonselective endothelin receptor antagonist, well established as a treatment in the pediatric population for idiopathic pulmonary hypertension. Its clinical benefit usually requires 3 months treatment, and its clinical effects have been mostly studied after 3 months of therapy [12, 17–21]. Sildenafil inhibits phosphodiesterase type 5 (PDE-5) in smooth muscle of the pulmonary vasculature. It has been shown to be beneficial in pediatrics for idiopathic pulmonary hypertension [22–25], persistent pulmonary hypertension of the newborn [26–28], and for pulmonary hypertension related to congenital heart disease [29–34]. 

Furthermore, approximately 7% [35] of children receiving a transplant require ventricular assist devices, which by unloading the left heart also lower pulmonary pressures associated with cardiomyopathies and left atrial hypertension. Numerous studies have proven beneficial effects of pre-OHT VAD placement for lowering PVR and allowing for transplantation [36–38].

This study reviews the initial experience with sildenafil and bosentan in addition to post-operative inhaled nitric oxide for pediatric cardiomyopathy patients with elevated pulmonary vascular resistance before and after orthotopic heart transplantation.

## 2. Methods

### 2.1. Patients

After obtaining Institutional Review Board approval, all patients undergoing OHT from 2002 to 2007 were retrospectively reviewed. During this time period, sildenafil and/or bosentan was used for all pediatric OHT patients with PVR > 6 WU indexed to body-surface area. Pre-operative and post-operative data was obtained for all patients including: age, weight, height, body-surface area, diagnosis, PVR (pre and post OHT), donor weight, resolution and recurrence of pulmonary hypertension, length of hospital stay, length of post-OHT medical treatment, graft rejection, deaths, echocardiographic data, and catheterization data. All PVR data are given in WU and are indexed to body surface area. Outcome data for 7 patients with elevated pre-operative PVR was compared to outcomes of 20 patients with normal pre-operative PVR transplanted during the same time period at UCLA Medical Center. The pre-OHT catheterization data closest to the time of transplant was compared to data at 1 week to 1 month, 3 months, and one-year post-OHT (see Table 1).

### 2.2. OHT/PVR Management

All of the patients with elevated PVR at pre-OHT catheterization were admitted to the ICU and treated with inotropes and diuretics; four of these patients underwent testing for response of PVR to administration of nitric oxide (NO). After OHT, immunosuppression consisted of tacrolimus (Prograf, Astellas, Deerfield, IL) mycophenolate mofetil (Cell-Cept Roche, Basel), and a prednisone taper. The steroid dosing was 2 mg/kg intravenous every eight hours times 3 doses, then 1 mg/kg divided twice a day weaned over two weeks to 1/2 mg/kg divided twice a day. Prednisone was weaned to off over the first year in low-risk patients without rejection. Prednisone was maintained at a low dose in presensitized patients with elevated panel reactive antibodies, and pulses were given to patients with rejection episodes. 

Bosentan (Actelion Pharmaceuticals, San Francisco, CA) or sildenafil (Pfizer, New York, NY) was used to treat pretransplant pulmonary hypertension. Treatment was begun after catheterization in all patients with PVR > 6, and length of treatment depended on waiting time for OHT. Bosentan was chosen for patients who had symptoms and a diagnosis greater than 3 months or lower than 1b transplant waiting list status while sildenafil was given to those with shorter expected diagnosis to transplant times. Patients were treated with either sildenafil and/or tracleer for an average of 56 days prior to receiving OHT (range 15–171 days). Sildenafil dosing was 0.5 mg/kg/dose orally three times a day, titrated to 1 mg/kg/dose as tolerated. Bosentan dosing was 62.5 mg orally twice a day for patients under 40 kg, and 125 mg for patients over 40 kg. Bosentan was not used post-OHT because of the potential for elevated blood levels due to interaction with tacrolimus. Liver function tests were checked monthly in patients receiving bosentan. Nitric oxide (NO) was used in all case patients in the immediate postoperative period in the intensive care unit as a prophylactic measure. NO was begun at doses of 5–10 ppm and weaned off prior to extubation. One patient with persistent pulmonary. hypertension after OHT was treated with inhaled iloprost (Ventavis. Actelion Pharmaceuticals, San Francisco, CA), 5 mcg six times per day.

### 2.3. Statistics

Statistical analysis was performed by comparing continuous variables using Student's *t*-test with Microsoft Excel (Microsoft Corp., Redmond, WA). *P*-values of less than  .05 were considered significant.

## 3. Results

### 3.1. Pre-OHT

The seven patients with pulmonary hypertension ranged from 4–18 years at the time of starting pulmonary vasodilator therapy. Four were females. Pre-operative diagnoses included four with dilated cardiomyopathy (DCM) and three with restrictive cardiomyopathy (RCM). The comparison group included 5 males and 15 females, ages 1–19, all of whom had pre-op PVR < 5 WU. Diagnoses of the comparison group included 19 with DCM, and one that developed cardiomyopathy after mitral valve replacement for Shone's complex. The mean PVR prior to transplantation in the 7 study cases was 11.0 ± 4.6 (range 6–22) WU, compared to mean PVR of 3.07 ± 0.9 WU (0.6–4.5) in the controls (*P* = .27 × 10^−6^).

Four of the patients with pulmonary hypertension were tested pre-OHT for PVR reactivity with oxygen and NO at catheterization. Three of these patients had <2 WU decrease in PVR, while one with RCM decreased from 13 WU to 8 WU. Three cases were not tested for PVR reactivity prior to OHT. 

Five of the cases with pulmonary hypertension (four with DCM and one with RCM) were treated pre-OHT with sildenafil (ranging from 10–87 days of treatment), and the three with RCM with bosentan (90–240 days treatment). One patient with RCM, an 18-year-old female, was treated pre-OHT with both medications. Three of the 7 pulmonary hypertension cases with DCM (including the one with PVR of 13 WU) were bridged to OHT with a ventricular assist device: ECMO (*n* = 2; for 3 and 10 days pre-OHT) or a biventricular assist device (*n* = 1; for 2.5 months pre-OHT).

### 3.2. Post-OHT

All 7 cases survived one year post-OHT. There were no readmissions for RV failure. The comparison group had a 90% survival at one year, with two deaths as a result of severe graft rejection (*P* = .8). Average hospital stay for the 7 case patients was longer than for the control group (31.3 days, versus 19 days for the controls). Measures of cardiac function were similar 1-2 weeks postoperatively with no significant difference in the following clinical data between cases and controls: thermodilution cardiac index (3.2 lpm/m^2^ versus 2.5 lpm/m^2^) (*P* = .14), pulmonary capillary wedge pressure (12 mmHg versus 13 mmHg) (*P* = .86), left ventricular ejection fraction estimated by *m*-mode and Simpson's rule with 2-dimensional echocardiography (62.5% versus 62%) (*P* = .64) (see Table 2).

**Figure 1:**
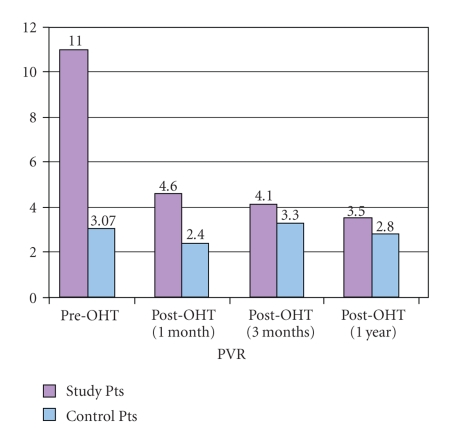


Mean PVR in the cases at one month post-OHT was 4.6 ± 1.8 WU (1.6–6.9 WU) compared to 2.4 ± 1.4 WU (0.5–6.9) in the comparison group (*P* = .002). At 3 months post-OHT, the mean PVR of the case group was 4.1 ± 2.6 (2.3–10) compared to 3.3 ± 1.9 (0.5–7) in the comparison group (*P* = .42). At the 3-month time point, four of the 7 pulmonary hypertension patients had PVR less than 4 WU. At one year, the mean PVR for the case patients was 3.5 ± 1.5 WU (2.6–5.4; *n* = 5) compared to 2.8 ± 1.8 WU (0.8–4.7), (*P* = .2) (see Figure 1). One control patient had a PVR at 1 and 3 months of 6.9 WU that decreased to below 3 WU by 6 months. This patient initially had a low-cardiac output requiring significant inotropic support that recovered during the second week post-OHT, but did not receive treatment for pulmonary hypertension.

Sildenafil treatment was initiated postoperatively in the four cases with pre-OHT minimally-reactive PVR. At 3 months post-OHT, these patients had a PVR of 3.6, 3.6, 5.9, and 7.5 WU. Sildenafil was weaned off by 6 months in 3 patients once the PVR was less than 3 WU. By one year, the patient with PVR of 10 WU at 3 months had a PVR of 5.4 WU. This patient had a pre-OHT diagnosis of RCM and was treated 240 days with bosentan preoperatively. There had been an 8-year interval between initial diagnosis and OHT. This patient was started on inhaled iloprost at 6 months post-OHT, in addition to sildenafil. At 2 years post-OHT, she had a PVR less than 2 WU and her iloprost and sildenafil were stopped. There were no observed interactions between sildenafil and iloprost with immunosuppressive medications, and no significant side-effects.

## 4. Discussion

The successful heart transplantation of seven pediatric cardiomyopathy patients with pulmonary hypertension associated with left heart failure is reported. All case patients had PVR > 6 WU at pre-OHT catheterization, tolerated medical treatments, and had no significant post-OHT graft failure. 

The elevated PVR in all of these cardiomyopathy patients was likely secondary to the direct effects of elevated left atrial pressures on the pulmonary vascular bed [39]. Improvement in pulmonary pressures post-OHT may be attributed to a decrease in left ventricular diastolic pressures with increased cardiac output of the functional left ventricle of the donor heart. However, during the time from development of the cardiomyopathy to heart transplantation, vascular remodeling of the pulmonary bed may occur and pulmonary hypertension may progress [40]. Pre-existing pulmonary hypertension is known to lead to increased mortality due to graft right heart failure [8, 9], with the peri-operative period being a high-risk time for pulmonary hypertensive episodes and right heart graft failure. In fact, a recent study looking at patients undergoing transplantation with pre-operative elevated PVR had a 75% incidence of post-operative RV failure, as compared to a 20% in those without elevated PVR [9]. This, we feel, makes a strong argument for the use of pulmonary vasodilators in patients with elevated pulmonary vascular resistance pretransplantation, and perhaps the use of post-operative inhaled nitric oxide and oral pulmonary vasodilators as well. 

As expected, patients with long standing pulmonary hypertension may take longer to recover a normal PVR after OHT. One patient described here went 9 years between the diagnosis of a restrictive cardiomyopathy and heart transplantation. She had persistently elevated PVR after OHT. This patient had persistent pulmonary hypertension up to 12 months post-OHT, despite normal left ventricular function and hemodynamics. By 2 years post-OHT, the pulmonary hypertension resolved and she is now off all pulmonary vasodilators. The other patients with a diagnosis of RCM had shorter periods with pulmonary hypertension prior to OHT, but required treatment 3–6 months after transplantation. Aside from the patient with RCM mentioned above, the patients with DCM and RCM did not have significant differences in recovery of PVR or hospital stay. In fact, two of the patients bridged with ECMO had post-OHT hospital stays longer than 1 month. The longer hospital stays were likely to have been a function of other postsurgical issues rather than elevated PVR.

Cardiomyopathy patients expected to have long waiting times for a donor were treated with bosentan. Sildenafil is more appropriate in the peri-operative period due to its more rapid effects on the pulmonary vasculature. We now use sildenafil, along with nitric oxide in the immediate post-operative period for patients with pre-existing pulmonary hypertension. Cyclosporine, a calcineurin inhibitor, is thought to raise the blood levels of bosentan, and thus increase the risk of liver damage [41]. Because tacrolimus is also a calcineurin inhibitor and a part of our standard immunosuppression regimen, we have not used bosentan post-OHT. We did not observe any side effects or adverse interactions between these medications and the immunosuppressive medications our patients were taking. Finally, mechanical support has been shown also to reduce PVR, making transplantation a successful option, and three of our patients with DCM and pulmonary hypertension were bridged to heart transplantation with assist devices. 

Limitations of this study include the small sample size and the retrospective nature of the study. Ideally, such a study would include patients with elevated PVR that did not receive pulmonary vasodilators for comparison to treatment cases; however, this has not been possible due to the small number of patients at a single institution. Additionally, the control patients who underwent heart transplantation during the same period as the study patients nearly all had dilated cardiomyopathy, whereas three of the seven study patients had a diagnosis of restrictive cardiomyopathy. Although all of our study patients did well posttransplantation, this is a distinction between our study and control patients worth noting. Three of our 7 study patients were not tested for PVR reactivity prior to starting of pulmonary vasodilator therapy, which is another consequence of the limited available data in this retrospective study. Furthermore, three of our 7 study patients were placed on an assist device (in addition to receiving pulmonary vasodilator therapy) prior to transplantation. Therefore, successful heart transplantation in these three patients cannot be completely attributed to placing them on pulmonary vasodilator therapy alone, and the role of ventricular assist devices in selecting patients with respect to successful heart transplantation needs to be investigated further. And finally, our study group consisted of seven patients with cardiomyopathy and pulmonary venous hypertension who ultimately received heart transplantation successfully with the aid of pulmonary vasodilators. The same, however, may not hold true for other conditions and other forms of pulmonary hypertension, and so further research is warranted on these patients and pulmonary vasodilator therapy. In summary, although this small retrospective study contains numerous limitations, recent pulmonary vasodilator therapies, in conjunction with other treatment modalities, present the prospect for heart transplantation in the pediatric population. More studies in the pediatric population, with a larger sample size and of a prospective nature, are required to study these promising therapies and their role in heart transplantation in the future.

## Figures and Tables

**Table 1:** 

Patients	Males	Females	Weight at	Pre-OHT	Days pre-OHT on	Pre-OHT	Pre-OHT	Pre-OHT Mean	Pre-OHT
			time of OHT (kg)	Dx	vasodilator tx	PVR	TPG	PAP (mmHg)	PCWP (mmHg)
Cases	3	4	30.8	3 RCM	56	11	15.3	45	28.3
			(11.6–53.7)	4 DCM	(15–171)	(6–22)	(9–21)	(30–57)	(20–38)

Controls	5	15	34.9	19 DCM	—	3.07	6.3	24.2	17.9
			(7–70)	1 Shone's Complex		(0.6–4.5)	(4–10)	(14–45)	(8–36)

**Table 2:** 

Patients	Post OHT	Post OHT (1-2 wk)	Post OHT (1-2 wk)	Post OHT (1-2 wk)
	Hospital stay (days)	C.I. (L/min/m^2^)	LV EF%	PCWP (mmHg)
Cases	31.3	3.2	62.5	12

Control	19	2.5	62	13
